# The Clinical Utility of the Child SCAT5 for Acute Concussion Assessment

**DOI:** 10.1186/s40798-022-00499-8

**Published:** 2022-08-13

**Authors:** Nicholas K. Erdman, Patricia M. Kelshaw, Samantha L. Hacherl, Shane V. Caswell

**Affiliations:** 1 Sports Medicine Assessment Research & Testing (SMART) Laboratory, Athletic Training Education Program, School of Kinesiology, George Mason University, 10890 George Mason Circle, Katherine Johnson Hall 221, MSN 4E5, Manassas, VA 20110 USA; 2 Virginia Concussion Initiative, George Mason University, Manassas, VA USA; 3 Advancing Healthcare Initiatives for Underserved Students (ACHIEVES) Project, George Mason University, Manassas, VA USA; 4 Brain Research and Assessment Initiative of New Hampshire (BRAIN) Laboratory, Department of Kinesiology, University of New Hampshire, Durham, NH USA

**Keywords:** Neurocognitive, Balance, Symptomology, Children, Diagnostic accuracy

## Abstract

**Background:**

The Child Sport Concussion Assessment Tool 5th Edition (Child SCAT5) was developed to evaluate children between 5 and 12 years of age for a suspected concussion. However, limited empirical evidence exists demonstrating the value of the Child SCAT5 for acute concussion assessment. Therefore, the purpose of our study was to examine differences and assess the diagnostic properties of Child SCAT5 scores among concussed and non-concussed middle school children on the same day as a suspected concussion.

**Methods:**

Our participants included 34 concussed (21 boys, 13 girls; age = 12.8 ± 0.86 years) and 44 non-concussed (31 boys, 13 girls; age = 12.4 ± 0.76 years) middle school children who were administered the Child SCAT5 upon suspicion of a concussion. Child SCAT5 scores were calculated from the symptom evaluation (total symptoms, total severity), child version of the Standardized Assessment of Concussion (SAC-C), and modified Balance Error Scoring System (mBESS). The Child SCAT5 scores were compared between the concussed and non-concussed groups. Non-parametric effect sizes ($r=\frac{Z}{\sqrt{n}}$) were calculated to assess the magnitude of difference for each comparison. The diagnostic properties (sensitivity, specificity, diagnostic accuracy, predictive values, likelihood ratios, and diagnostic odds ratio) of each Child SCAT5 score were also calculated.

**Results:**

Concussed children endorsed more symptoms (*p* < 0.001, $r$=0.45), higher symptom severity (*p* < 0.001, $r$=0.44), and had higher double leg (*p* = 0.046, $r$=0.23), single leg (*p* = 0.035, $r$=0.24), and total scores (*p* = 0.022, $r$=0.26) for the mBESS than the non-concussed children. No significant differences were observed for the SAC-C scores (*p’s* ≥ 0.542). The quantity and severity of endorsed symptoms had the best diagnostic accuracy (AUC = 0.76–0.77), negative predictive values (NPV = 0.84–0.88), and negative likelihood ratios (-LR = 0.22–0.31) of the Child SCAT5 scores.

**Conclusions:**

Clinicians should prioritize interpretation of the symptom evaluation form of the Child SCAT5 as it was the most effective component for differentiating between concussed and non-concussed middle school children on the same day as a suspected concussion.

## Key Points

- Concussed middle school children endorsed more symptoms, reported greater symptom severity, and had worse balance performance as compared to middle school children who were not diagnosed with a concussion on the same day as the suspected injury.
- Healthcare professionals should prioritize the interpretation of the symptom evaluation form of the Child SCAT5 as it was the most effective component of the Child SCAT5 for differentiating between concussed and non-concussed middle school children when administered on the same day as a suspected concussion.
- Highly variable diagnostic properties were observed for the individual assessment of the symptom, neurocognitive, and balance components of the Child SCAT5 when administered to middle school children on the same day as a suspected concussion.

## Background

There are approximately 12.3 million middle school (grade level 6–8) age children in the United States and an estimated 36% (≈4.4 million) participate in organized intramural or interscholastic sport annually [1, 2]. Participation in organized sport for children under 14 years of age is the leading cause of concussion (43%)[3] and has been associated with a six times greater risk of concussion as compared to other leisure physical activities [4]. Limited on-site medical coverage of school-sponsored sports [5] contributes to a majority (86%) of children seeking medical care from healthcare professionals in direct access settings (e.g., emergency department, outpatient clinics) [6, 7] where follow up visits are uncommon (1–3%) [8–10].

Several governing bodies [11–14] recommend the implementation of a multi-modal assessment for the evaluation of children following a suspected concussion. The Sport Concussion Assessment Tool 5th edition (SCAT5) [15] and the Child Sport Concussion Assessment Tool 5th Edition (Child SCAT5)[16] are two of the most commonly used multi-modal assessments by licensed healthcare professionals for the acute evaluation and clinical management of concussion [17–20]. The Child SCAT5 is a modified version of the SCAT5 that was developed for administration to children between 5 and 12 years of age and consists of a symptom evaluation form, the child version of the Standardized Assessment of Concussion (SAC-C), and the modified balance error scoring system (mBESS) [15]. Previous literature has demonstrated poor psychometric (e.g., test–retest reliability) and diagnostic properties (e.g., sensitivity, specificity, predictive value, likelihood ratios) for the individual components of prior iterations of the SCAT [16, 21–40] and the Child SCAT 3rd Edition (Child SCAT3) [41–44]. Despite consensus recommendation and wide adoption, scant evidence exists regarding the psychometric and diagnostic properties of the Child SCAT5 [45–47].

The lack of empirical evidence regarding the Child SCAT5 necessitates that healthcare professionals rely upon their subjective interpretation of Child SCAT5 scores to inform acute clinical management. An approach which may contribute to misdiagnosis and inconsistent treatment for those children acutely evaluated for concussion using the Child SCAT5 [48]. Therefore, the purpose of our study was to evaluate for differences and assess the diagnostic properties of Child SCAT5 scores on the same day as a suspected concussion among middle school children who were (“concussed”) or were not (“non-concussed”) clinically diagnosed with a concussion. We hypothesized that (i) the concussed children would endorse significantly more symptoms and report a significantly higher symptom severity as compared to the non-concussed children; (ii) the concussed children would score lower on the SAC-C and commit significantly more errors on the mBESS as compared to the non-concussed children; and (iii) the symptom evaluation (total symptoms endorsed, symptom severity) would demonstrate better diagnostic properties than the SAC-C or mBESS on the same day as the suspected concussion.

## Methods

### Design and Settings

The Advancing Healthcare Initiatives for Underserved Students (ACHIEVES) Project provided on-site medical care to sixteen middle schools within a large socio-demographically diverse school district in Virginia, USA [49]. The George Mason University Institutional Review Board approved the construction of the deidentified database for research purposes and waived assent and consent. All participants in our study competed in school-sponsored sports between the 2017–2018 and 2019–2020 academic years.

### Participants

**Fig. 1:**
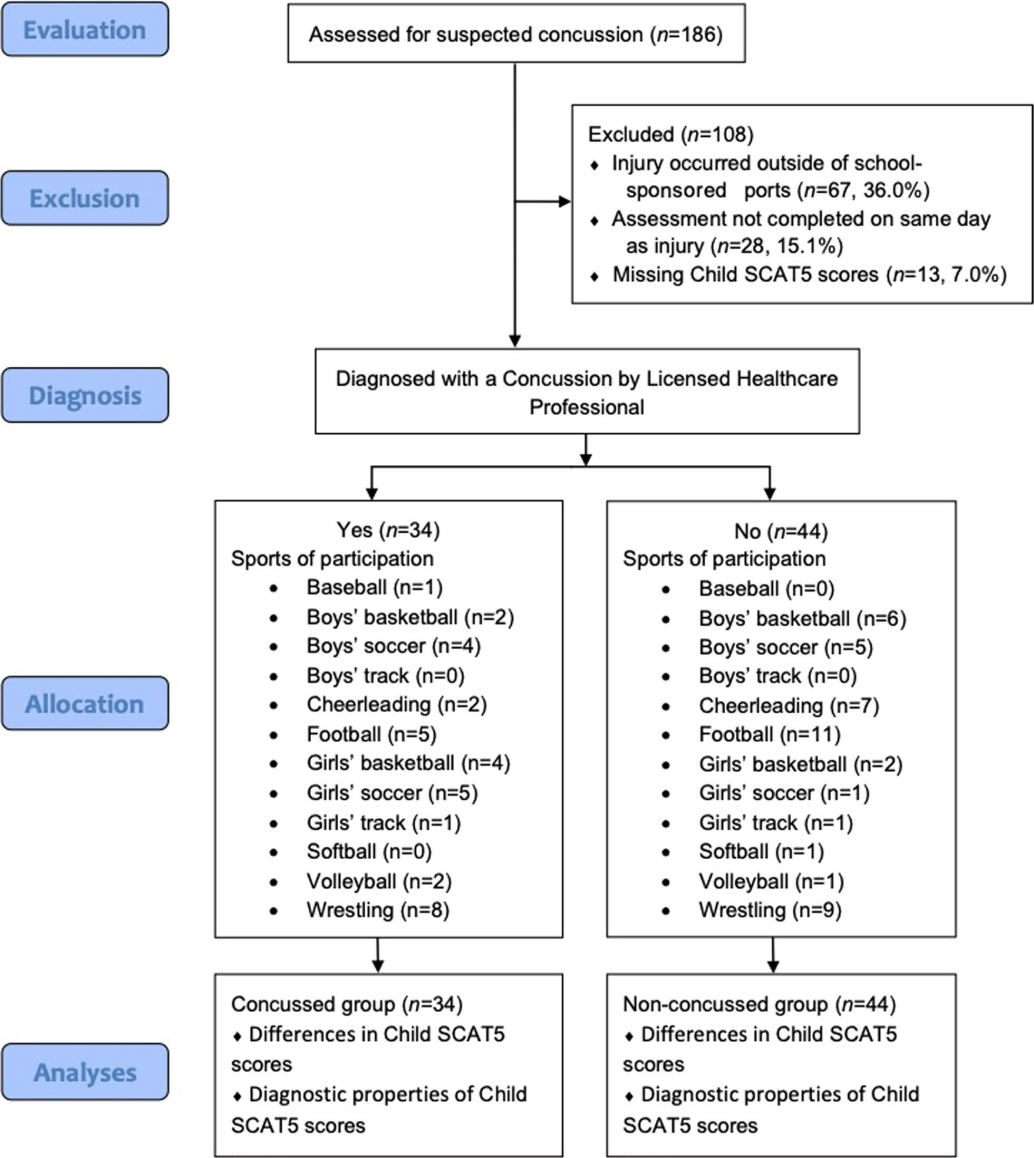
Flow chart for participant evaluation and analyses

Our sample originally consisted of 186 (103 concussed, 83 non-concussed) middle school children that were evaluated for a suspected concussion (Fig. 1). Exclusion criteria were set to ensure that injury and assessment data were available from the concussion assessment that was administered at the time of the suspected concussion. Participants were excluded if their suspected concussion occurred outside of sport, the initial assessment was not completed on the same day as the suspected concussion, or there were any missing data elements from the Child SCAT5 (Fig. 1). After applying all of our exclusion criteria, our final sample consisted of 78 (34 concussed, 44 non-concussed) middle school children that were evaluated for a suspected concussion while participating in school-sponsored sports. Certified athletic trainers administered the Child SCAT5 to all middle school children, up to age 13, participating in sport. The reason for this was two-fold: (i) to be consistent with prior studies on the Child SCAT3[42, 50] and Child SCAT5[46, 47] that include children up to age 13, and (ii) children could have been baseline measured with the Child SCAT5 at age 12 and then could potentially be injured at age 13; rather than change the assessment to the SCAT5 for that child, the athletic trainers used the Child SCAT5 to avoid the complexities of interpreting two distinct assessment tools.

### Testing Procedures

Consistent with Virginia Code (§ 22.1–271.5)[51] and the school system’s concussion management protocol, any middle school child exhibiting signs or symptoms consistent with a concussion was immediately removed from participation in school-sponsored sport and evaluated by the school’s certified athletic trainer. The Child SCAT5 was administered by certified athletic trainers, via pen and paper, using the standardized instructions provided for each component of the Child SCAT5 as published elsewhere [16, 46, 47]. The certified athletic trainers utilized their clinical judgement in respect to time, resources, and location to determine the most appropriate environment to administer the Child SCAT5 following a suspected concussion while limiting potential distractions and ensuring the safety of the student-athlete. All assessments were conducted on the day of the suspected concussion, operationally defined as having matching “date-of-injury” and “date-of-assessment” documentation in the electronic medical records. The definition of a concussion was consistent with the most recent international consensus statement on concussion in sport [11]. Regardless of their Child SCAT5 scores, the children were not permitted to return to sport participation on the same day as the suspected concussion which is in alignment with international consensus statements, several governing bodies, and Virginia Code.[11–14, 51, 52]. The cases included in our study were ascertained from deidentified electronic medical records. Cases were allocated into groups by the research team dependent on whether the child was clinically diagnosed (i.e., “concussed”) or was not clinically diagnosed (i.e., “non-concussed”) as having a concussion by the respective certified athletic trainer.

### Outcomes

The administration and scoring of each component of the Child SCAT5 (symptom evaluation, child version of the Standardized Assessment of Concussion [SAC-C], and the modified version of the balance error scoring system [mBESS]) are described in detail elsewhere [16, 46, 47]. For the symptom evaluation, the total number of symptoms endorsed (range = 0–21) and the total symptom severity (range = 0–63) endorsed by the children were calculated. For the SAC-C, points earned for the 5-word immediate memory (range = 0–15 points), concentration (range = 0–6 points), and delayed recall (range = 0–5 points) domains were recorded and summed to calculate a composite score (range = 0–26 points). For the mBESS, errors committed (range = 0–10) during each stance (double leg, single leg, tandem) were recorded and summed to calculate the total score (range = 0–30). Lower scores on the SAC-C and higher scores on the mBESS were indicative of worse performance on the respective components of the Child SCAT5.

### Statistical Analyses

Nonparametric analyses were performed due to the non-normal distribution (Shapiro–Wilk = 0.78–0.94, *p*’s < 0.05) of the Child SCAT5 scores. Mann–Whitney U-tests were used to assess for differences between the concussed and non-concussed children for each Child SCAT5 score. Nonparametric effect sizes[53] ($r=\frac{Z}{\sqrt{N}}$) were calculated and interpreted as small (*r* = 0.10–0.30), moderate (*r* = 0.30–0.50), or large (*r* ≥ 0.50) [54].

Receiver operator curve analyses were performed to calculate the diagnostic accuracy (area under the curve [AUC]) of each Child SCAT5 score [21, 24, 25, 55–58]. Youden’s Index (*J*) [59] was calculated to determine the cutoff score that optimized the combination of sensitivity (Sn) and specificity (Sp) for each Child SCAT5 score [60–63]. Values closer to 1.0 for the Youden Index are indicative of a greater combination of sensitivity and specificity [59].

The sensitivity and specificity values of each Child SCAT5 score were used to calculate their positive (PPV) and negative (NPV) predictive values, positive (+ LR) and negative (-LR) likelihood ratios, and diagnostic odds ratios (DOR). Positive and negative predictive values that are closer to 1.0 are indicative of a more valid test result [64]. Higher positive likelihood ratios indicate a greater likelihood that the patient has the test condition (e.g., concussed) while lower negative likelihood ratios indicate a greater likelihood that the patient does not have the test condition (e.g., not concussed) [65]. Higher diagnostic odds ratios indicate that the test result has a greater ability to differentiate between patients with (e.g., concussed) and without (e.g., non-concussed) the test condition. All analyses were performed using SPSS (Version 27, IBM Corp., NY, USA). Alpha was set a priori at *p* < 0.05.

## Results

Our sample consisted of 34 concussed (21 male [62%], age = 12.8 ± 0.86 years) and 44 non-concussed (31 male [70%], age = 12.4 ± 0.76 years) middle school children who participated in a variety of school-sponsored sports including football, wrestling, baseball, softball, volleyball, cheerleading, basketball, soccer, and track (Fig. 1). The participants in the concussed and non-concussed groups were not significantly different across any demographic variable (*p*’s > 0.05). Please see Table 1 for participant demographic and pre-existing health condition information.

**Table 1:** Descriptive statistics for our study sample

	Total	Concussed	Non-Concussed
	N = 78	*n* = 34	*n* = 44
Age M (SD)	12.6 (0.83)	12.8 (0.86)	12.4 (0.76)
Grade M (SD)	6.9 (0.74)	7.2 (0.72)	6.7 (0.69)
*Gender (n, %)*			
Boys	52 (66.7%)	20 (58.8%)	30 (68.2%)
Girls	26 (33.3%)	14 (41.2%)	14 (31.8%)
English primary language spoken at home (n, %)	68 (87.2%)	26 (76.5%)	42 (95.5%)
*Previous concussion history*			
Zero prior concussions (n, %)	66 (84.6%)	27 (79.4%)	39 (88.6%)
1 prior concussion (n, %)	22 (14.1%)	7 (20.6%)	4 (9.1%)
2 or more prior concussions (n, %)	1 (1.3%)	0 (0.0%)	1 (2.3%)
*Pre-Existing Health Conditions*			
Hospitalized for a head injury (n, %)	17 (7.8%)	1 (2.9%)	3 (6.8%)
Headache disorder/migraines (n, %)	8 (3.7%)	2 (5.9%)	1 (2.3%)
Learning disability/dyslexia (n, %)	2 (0.9%)	0 (0.0%)	0 (0.0%)
ADHD (n, %)	14 (6.4%)	2 (5.9%)	3 (6.8%)
Psychiatric condition (n, %)	6 (2.7%)	0 (0.0%)	1 (2.3%)

**Fig. 2:**
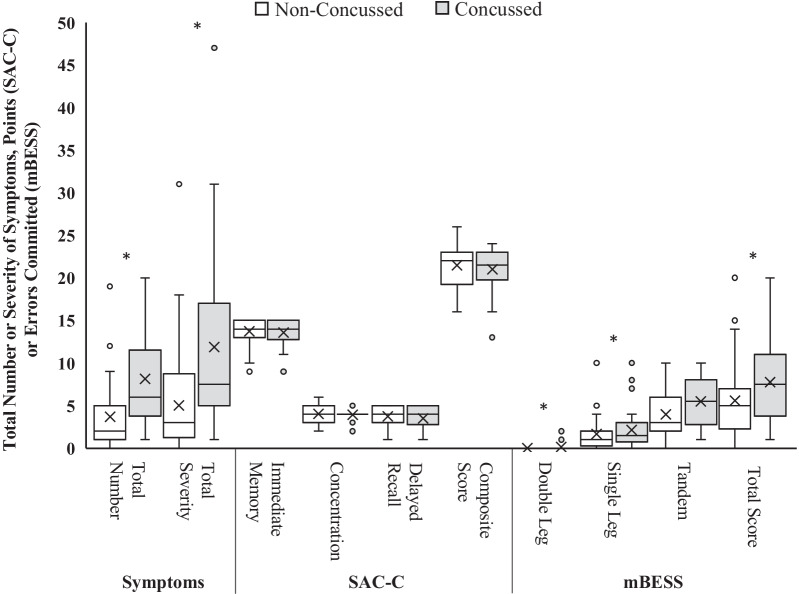
Group comparisons for the Child Sport Concussion Assessment Tool 5th Edition (Child SCAT5) scores. *Note* Boxes represent the first, second, and third quartile values, error bars represent maximum (third quartile + 1.5*Interquartile Range) and minimum (third quartile – 1.5* Interquartile Range) values, X = group mean, SAC-C = child version of the Standardized Assessment of Concussion, mBESS = modified balance error scoring system. *P < .05

**Fig. 3:**
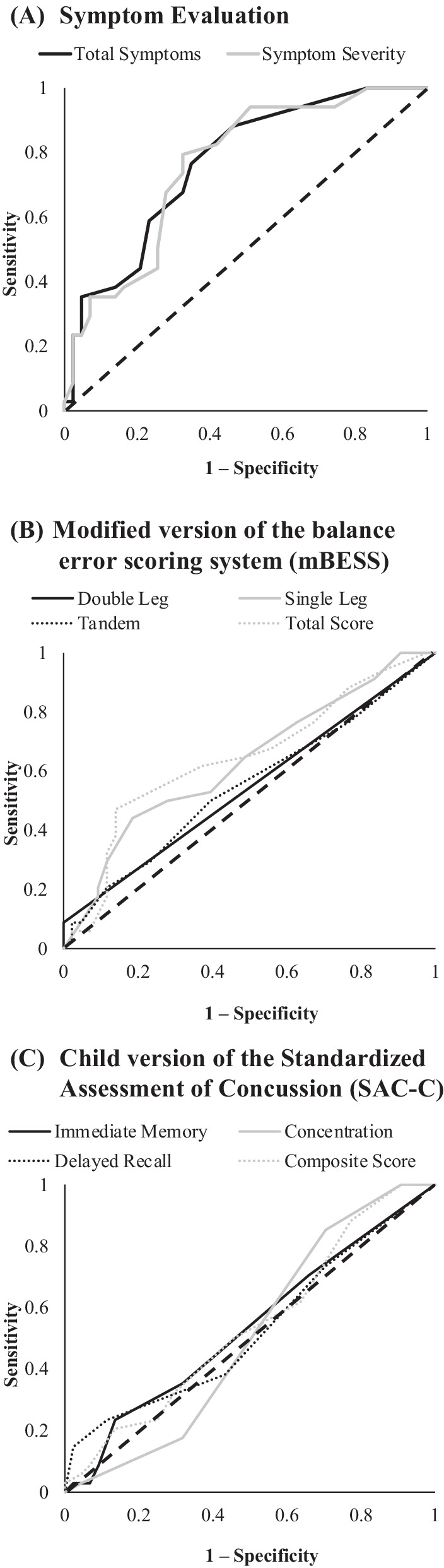
Receiver operator curves for the Child Sport Concussion Assessment Tool 5^th^ Edition (Child SCAT5) scores. Note: Dashed line is reference line demonstrating equitable sensitivity and specificity

The concussed children endorsed significantly more symptoms (median = 6.0 [IQR = 4–12] vs. median = 2.0 [IQR = 1–5], *p* < 0.001, $r$=0.45) and reported greater symptom severity (median = 7.5 [IQR = 5–17] vs. median = 3.0 [IQR = 1–9], *p* < 0.001, $r$=0.44) than the non-concussed group (Table 2, Fig. 2). The total number and severity of endorsed symptoms resulted in the highest diagnostic accuracy (AUC = 0.76–0.77) and negative predictive values (NPV = 0.84–0.88) and the lowest negative likelihood ratios (− LR = 0.22–0.31) of the Child SCAT5 scores (Table 3, Fig. 3A).

**Table 2:** Descriptive statistics for the Child Sport Concussion Assessment Tool – 5^th^ Edition (Child SCAT5) scores

	Concussed					Non-concussed					Group Comparisons	
Child SCAT5 Component	Range	M	SD	Md	IQR	Range	M	SD	Md	IQR	P	*r*
*Symptoms*												
Total Number	1–20	8.0	5.50	6.0	4–12	0–19	3.7	3.97	2.0	1–5	< .001	0.45
Total Severity	1–47	12.1	10.47	7.5	5–17	0–31	5.0	6.01	3.0	1–9	< .001	0.44
*SAC-C*												
Immediate Memory	6–15	13.6	1.46	14.0	13–15	9–15	13.7	1.47	14.0	13–15	.582	0.06
Concentration	2–6	3.9	0.65	4.0	4–4	2–6	4.0	1.02	4.0	3–5	.896	0.01
Delayed Recall	2–5	3.5	1.38	4.0	3–5	1–5	3.7	1.06	4.0	3–5	.684	0.05
Composite Score	13–24	21.0	2.61	21.5	20–23	16–26	21.5	2.48	22.0	19–23	.542	0.07
*mBESS*												
Double Leg	0–2	0.2	0.50	0.0	0–0	0–0	0.0	0.00	0.0	0–0	.046	0.23
Single Leg	1–10	5.5	3.12	5.5	3–8	0–10	4.0	2.85	3.0	2–6	.035	0.24
Tandem Leg	0–10	2.2	2.36	1.5	1–3	0–10	1.6	1.83	1.0	0–2	.398	0.10
Total Score	1–20	7.8	4.53	7.5	4–11	1–20	5.6	4.17	5.0	2–7	.022	0.26

SAC-C = Standardized Assessment of Concussion—Child Version, mBESS = Modified Balance Error Scoring System, M = mean, SD = standard deviation, Md = median, IQR = interquartile range (25^th^ to 75^th^ percentile), *r* = effect size

**Table 3:** Diagnostic properties for the Child Sport Concussion Assessment Tool 5th edition (Child SCAT5) scores

Outcome Measure	Cutoff Score	Sn	Sp	*J*	AUC	PPV	NPV	+ LR	-LR	DOR
*Symptoms*										
Total Endorsed	≥ 3 symptoms	0.88	0.54	0.47	0.77	0.54	0.88	1.9	0.22	8.6
Total Severity	≥ 5 points	0.79	0.67	0.42	0.76	0.6	0.84	2.44	0.31	7.97
*SAC-C*										
Immediate Memory	≤ 12 points	0.24	0.86	0.1	0.54	0.51	0.65	1.73	0.89	1.95
Concentration	≤ 4 points	0.85	0.3	0.15	0.51	0.43	0.77	1.21	0.5	2.43
Delayed Recall	≤ 1 point	0.15	0.98	0.12	0.53	0.8	0.65	6.39	0.87	7.32
Composite	≤ 18 points	0.88	0.23	0.11	0.54	0.41	0.76	1.14	0.52	2.19
*mBESS*										
Double Leg	≥ 1 error	0.09	1	0.09	0.54	0.98	0.64	88	0.91	96.39
Single Leg	≥ 7 errors	0.44	0.81	0.11	0.55	0.59	0.7	2.37	0.69	3.45
Tandem	≥ 2 errors	0.5	0.61	0.26	0.63	0.44	0.66	1.27	0.83	1.53
Total	≥ 9 errors	0.47	0.86	0.33	0.64	0.67	0.73	3.36	0.62	5.47

SAC-C = child version of the Standardized Assessment of Concussion, mBESS = modified version of the balance error scoring system, Sn = sensitivity, Sp = specificity, *J* = Youden Index, AUC = area under the curve, PPV/NPV = positive and negative predictive values, + LR/-LR = positive and negative likelihood ratios, DOR = diagnostic odds ratios

None of the SAC-C scores were significantly different between the concussed and non-concussed children (*p’s* ≥ 0.54, Table 2). The SAC-C had the lowest diagnostic accuracy values (AUC = 0.51–0.54) of the Child SCAT5 scores (Table 3, Fig. 3B).

The concussed children committed significantly more errors in the double leg (median = 0.0 [IQR = 0–0] vs median = 0.0 [IQR = 0–0], *p* = 0.046, $r$=0.23) and single leg (median = 5.5 [IQR = 3–8] vs median = 3.0 [IQR = 2–6], *p* = 0.035, $r$=0.24) stances of the mBESS as compared to the non-concussed children (Table 2). Significantly higher total scores on the mBESS (median = 7.5 [IQR = 4–11] vs median = 5.0 [IQR = 2–7], *p* = 0.022, $r$=0.26) were observed for the concussed children as compared to the non-concussed children (Table 2). Committing at least one error in the double leg stance resulted in the highest specificity (Sp = 1.00) but the lowest sensitivity (Sn = 0.09; Table 3) values. A total mBESS score of at least nine errors had higher diagnostic accuracy (AUC = 0.64) than interpretation of the individual stances (AUC = 0.54–0.63; Table 3, Fig. 3C).

## Discussion

Despite limited empirical evidence, the Child SCAT5 is recommended for use by healthcare professionals to assist with the clinical diagnosis of concussion in children [11–14]. This study was conducted to better understand the utility of the Child SCAT5 on the day of a suspected concussion. Our findings provide evidence that the symptom component was clinically useful, whereas the SAC-C and mBESS components of the Child SCAT5 were not useful for differentiating between concussed and non-concussed middle school children on the same day as a suspected concussion. As many children receive acute care for suspected head injuries in busy direct access settings (e.g., emergency departments or outpatient clinics), findings from our study suggest that healthcare professionals in these settings should prioritize the interpretation of the symptom evaluation components of the Child SCAT5 during the acute clinical evaluation of middle school age children with a suspected concussion.

Self-reported symptomology has been a cornerstone of concussion evaluation and remains the key component for informing clinical judgement [11]. Using the Child SCAT3 in an emergency department setting, Babl and colleagues [44], demonstrated that concussed children endorsed significantly more symptoms and reported greater symptom severity than non-concussed children. Similarly, using the Child SCAT5 for acute evaluation, we found that concussed children endorsed a significantly greater total number (median = 6.0 [IQR = 4–12] vs. median = 2.0 [IQR = 1–5]) and severity (median = 7.5 [IQR = 5–17] vs. median = 3.0 [IQR = 1–9] of symptoms than non-concussed children. Additionally, based on our calculated cutoff scores, children who endorsed less than four symptoms or reported a severity less than six points on the same day as the suspected concussion were less likely (− LR = 0.22–0.31) to be clinically diagnosed with a concussion. A notable finding of our study is that all children suspected of sustaining a concussion presented acutely with at least one symptom. However, the children that were clinically diagnosed with a concussion, endorsed a considerably greater number and severity of symptoms than those who were not diagnosed. This reveals the following complexities: (i) the lack of specificity of the individual symptom items in the Child SCAT5 may contribute to symptom endorsement among children that in fact do not have a concussion; (ii) however paradoxically, the total number and severity of symptoms endorsed are likely the most valuable clinical measure of the Child SCAT5 in terms of diagnostic accuracy. Thus, in direct-access settings, the endorsed number and severity of symptoms on the Child SCAT5 should inform clinical decision making. However, more research is necessary to investigate which individual symptoms or symptom clusters of the Child SCAT5 are the most clinically useful for the acute assessment of concussion in children.

The concussed children in our study did not perform significantly different on any component of the SAC-C as compared to the non-concussed children on the same day as the suspected concussion. Additionally, we detected no significant differences in the SAC-C composite scores between the concussed and non-concussed children and the values for each group were within the “broadly normal” interpretation of normative reference values for this population [46]. The lack of a significant difference between concussed and non-concussed children diverges from previous research that evaluated older athletic populations (using the SAC) [22, 23, 66–69]. These differences may be attributed to different age-ranges of the participants (e.g., high school and collegiate athletes) and different ranges of composite scores for the SAC (range = 0–30) and SAC-C (range = 0–26) [15]. Furthermore, the SAC and SAC-C were developed for acute and post-acute assessments of concussion, however, recent evidence suggests that the clinical utility of the SAC may decline after 3–5 days post-concussion [70]. Presently, it is unclear when the optimal clinical assessment time interval is for post-concussion interpretation of the SAC-C.

Concussed children in our study had significantly higher mBESS total scores (median = 7.5 [IQR = 4–11]; 7.8 ± 4.5 errors) than non-concussed children (median = 5.0 [IQR = 2–7]; 5.6 ± 4.2 errors) and also slightly higher than the normative reference values for this population (5.0 ± 3.7 errors) [46]. These findings are consistent with reports that concussed high school and college athletes perform worse on the BESS than non-concussed athletes [22, 23, 66–69]. However, the small effect sizes (*r* = 0.23–0.26) for the concussed versus non-concussed comparisons observed in our study suggest that these statistical differences may not be clinically meaningful. For example, the mean difference of approximately two errors on the mBESS total score between the concussed and non-concussed groups aligns well with expected variability in mBESS performance as supported by previously established values for determining a clinically meaningful change [22, 47, 71]. Thus, the differences observed between the concussed and non-concussed groups may be due to repeated administration of the mBESS rather their medical status. Additionally, the diagnostic accuracy of the individual stances of the mBESS as well as the total score were low (AUC = 0.54–0.64) further suggesting limited clinical utility of the mBESS when acutely administered to middle school children suspected of having a concussion.

When examining the least accurate diagnostic components of the Child SCAT5 (i.e., the SAC-C and mBESS), the highest positive predictive values and likelihood ratios were observed for interpretation of the double leg stance of the mBESS followed by the delayed recall domain of the SAC-C. However, inordinately low thresholds for a positive test result (e.g., diagnosed concussion) for the double leg stance of the mBESS (≤ 1 error) and the delayed recall domain of the SAC-C (≤ 1 point) yielded high specificity values (Sp = 0.98–1.00) which artificially elevated the calculated positive predictive values and likelihood ratios. Therefore, we caution healthcare professionals from independent interpretation of the double leg stance of the mBESS or the delayed recall domain of the SAC-C in their clinical decision-making at this time. Moreover, evidence of poor reliability of the SAC-C and mBESS components of the Child SCAT5 further demonstrates the limited clinical utility of these measures within this patient demographic [47]. Future research should investigate alternative assessment tools (e.g., tandem gait test[72]) or strategies (e.g., the dual task paradigm[73]) for the acute evaluation of children following a suspected concussion.

Overall, our findings support those of previous research [23, 44, 66, 68] which suggest that the symptom evaluation has the best combination of diagnostic properties and is the most effective component of the Child SCAT5 for differentiating between concussed and non-concussed children. Using similar methodology, Babl and colleagues [44], observed diagnostic accuracy values for Child SCAT3 scores that are comparable to those observed for the Child SCAT5 in our study [44]. While similar findings between Child SCAT3 and the Child SCAT5 affirms the value of symptoms, jointly these findings may also suggest that other components of the Child SCAT5 have not improved and that further refinement is necessary to improve its clinical utility for the acute assessment of concussion in children.

Healthcare professionals in direct access settings (e.g., emergency department, outpatient clinics) may be the first to evaluate a child following a suspected concussion [6] and commonly do not have preinjury (i.e., baseline) scores. As such it is vital that healthcare professionals are equipped with age-appropriate assessment tools that can effectively differentiate between those children who are and are not concussed in order to appropriately inform patient care. Recommendations based upon empirical data that may assist healthcare professionals with clinical interpretation of the Child SCAT5 have been published [46, 47]. These include normative reference values which account for demographic characteristics (e.g., gender and age) [46], as well as psychometric information about the Child SCAT5 [47]. In conjunction with our findings, these studies offer guidance on using the Child SCAT5 to support clinical decision-making when acutely evaluating children with a suspected concussion.

We recognize that our study is not without limitations. We analyzed data gathered from children (age 10–13 years) that participated in middle school-sponsored sports within a single public school division in Virginia, which limits the generalizability of our findings to all children for which the Child SCAT5 may be used. However, these data were derived from middle schools having diverse socio-demographic student profiles (71.5% racial or ethnic minority; 47% economically disadvantaged) which is perhaps a more representative sample of the total middle school age population than previously studied [49]. Another limitation is our inability to control for the variability in the time between the removal from sport and the concussion evaluation. However, all children were administered the Child SCAT5 on the same day as the suspected concussive event. More research is needed to better understand the optimal time interval using the Child SCAT5 in acute situations. Further, the Child SCAT5 incorporates a parent symptom report, however, we were unable to include these data in our study due to inconsistent access to parents/guardians within this cohort. Future research should investigate the clinical utility of parent symptom reporting outcomes of the Child SCAT5 following a suspected concussion. Finally, we utilized the 5-word immediate memory list for the SAC-C score generation. The clinical utility of the 10-word list, particularly for children who may need a more challenging task than the 5-word list, should be investigated.

## Conclusion

The concussed middle school children endorsed more symptoms and reported greater symptom severity on the same day as the suspected concussion than children who were not diagnosed with a concussion. Furthermore, the total number and severity of the endorsed symptoms, on the Child SCAT5, resulted in the greatest combination of diagnostic properties as supported by the best sensitivity, diagnostic accuracy, negative predictive values, and negative likelihood ratios. Both the SAC-C and the mBESS failed to detect clinically meaningful differences between the concussed and non-concussed children and also had highly variable diagnostic properties. Collectively, our findings suggest that healthcare professionals working in direct access settings should prioritize symptom evaluation as it was the most effective component of the Child SCAT5 for the acute assessment of a child with a concussion.

## Data Availability

The datasets generated during and/or analyzed during the current study are available from the corresponding author on reasonable request.
